# Coronary Artery Calcification in Patients with Radiographic Axial Spondyloarthritis: A Comparative Study with Matched Controls in Southwestern Sweden

**DOI:** 10.3390/jcdd12080305

**Published:** 2025-08-12

**Authors:** Erik Hulander, Anna Deminger, Sofia Enegren, Magnus Hallström, Caroline Feldthusen, Erika Fagman, Oskar Angerås, Tatiana Zverkova Sandström, Mats Geijer, Helena Forsblad-d’Elia

**Affiliations:** 1 Department of Rheumatology and Inflammation Research, Sahlgrenska Academy, University of Gothenburg, 40530 Gothenburg, Sweden; 2 Clinical Nutrition Unit, Region Västra Götaland, Sahlgrenska University Hospital, 43145 Gothenburg, Sweden; 3 Department of Rheumatology, Region Västra Götaland, Sahlgrenska University Hospital, 41346 Gothenburg, Sweden; 4 Department of Occupational Therapy and Physiotherapy, Region Västra Götaland, Sahlgrenska University Hospital, 41685 Gothenburg, Sweden; 5 Department of Radiology, Region Västra Götaland, Sahlgrenska University Hospital, 41685 Gothenburg, Sweden; 6 Department of Radiology, Sahlgrenska Academy, University of Gothenburg, 41345 Gothenburg, Sweden; 7 Department of Cardiology, Region Västra Götaland, Sahlgrenska University Hospital, 41345 Gothenburg, Sweden; 8 Department of Molecular and Clinical Medicine, Sahlgrenska Academy, University of Gothenburg, 40530 Gothenburg, Sweden; 9 Department of Clinical Sciences, Lund University, 22100 Lund, Sweden

**Keywords:** radiographic axial spondyloarthritis, coronary artery calcification, cardiovascular disease risk, chronic inflammation

## Abstract

Radiographic axial spondyloarthritis (r-axSpA) is associated with increased cardiovascular disease (CVD) risk. The coronary artery calcification (CAC) score, an atherosclerosis burden indicator that predicts CVD risk, is not well studied in r-axSpA. This study investigates CAC scores in patients with r-axSpA compared to controls without rheumatic disease and factors associated with CAC scores in r-axSpA patients. Fifty-eight r-axSpA patients from southwestern Sweden were assessed cross-sectionally using clinical disease measures, physical function, spinal mobility, lipid profiles, inflammation markers, and long-term time-averaged C-reactive protein (CRP). Four controls per patient were selected from the Swedish CArdioPulmonary bioImage Study (SCAPIS). CAC was scored on cardiac computed tomography (CT) using the Agatston method. The presence of CAC in the right coronary artery (RCA) was higher in patients compared to controls. However, no significant difference in total CAC scores was observed between r-axSpA patients and controls, despite numerically higher total CAC scores in patients. In r-axSpA patients, CAC scores correlated positively with time-averaged CRP, reduced physical function, and impaired spinal mobility. These findings suggest that chronic inflammation may contribute to coronary calcification and CVD risk in r-axSpA, highlighting the need for effective anti-inflammatory treatments. Further research is warranted to explore the association between coronary calcification, spinal immobility, and limitations in physical function.

## 1. Introduction

Radiographic axial spondyloarthritis (r-axSpA) is one of the most common chronic inflammatory rheumatic diseases, primarily affecting the spine and sacroiliac joints, and is characterized by chronic back pain and stiffness. The disease usually manifests early in life (typically in the 20s), is more common in males than females, and leads to significant reductions in quality of life due to physical function impairments, fatigue, and high disease activity [1]. Patients with r-axSpA face higher mortality and an elevated risk of cardiovascular disease (CVD) compared to the general population [2]. Traditional risk factors, as well as chronic inflammation, influence the risk of CVD. The coronary artery calcification score (CAC score) is an established instrument that identifies calcification in the coronary arteries and can be used in risk prediction for CVD [3]. However, knowledge of CAC scores in patients with r-axSpA is limited.

Therefore, the objective of this study was to examine if the CAC score burden differs between patients with r-axSpA and matched controls and to investigate factors associated with the CAC score in the patients.

## 2. Materials and Methods

Patients with confirmed diagnosis (ICD-10 M45.9) of r-axSpA (modified NY criteria [4]) were recruited in southwestern Sweden (Sahlgrenska University Hospital, Södra Älvsborg Hospital, and Alingsås Hospital) in 2009, as previously described elsewhere [5]. Inclusion criteria were age >18 years and the ability to understand the Swedish language. Exclusion criteria were pregnancy, dementia, psoriasis, or inflammatory bowel disease. The present study is based on data from the latest follow-up, in a planned prospective cohort study that began in 2022 and was completed in 2023, which included 141 patients in the long-term outcome ankylosing spondylitis (LOAS) study.

Laboratory measures of inflammation (high-sensitivity C-reactive protein (hsCRP) and erythrocyte sedimentation rate (ESR)) were analyzed using standard clinical laboratory procedures. Patient records were searched for historical C-reactive protein (CRP) values; the first value of each year deemed unrelated to infection or any other emergency was recorded. Values from the year 2009 until and including the last follow-up were collected, and a time-averaged CRP value was calculated. The number of collected values (a combination of hsCRP and CRP) for each patient varied between 4 and 14, with a median of 10 measurements. Clinical disease measures (higher value indicating a worse outcome) of disease activity (the Ankylosing Spondylitis Disease Activity Score with CRP (ASDAS) and the Bath Ankylosing Spondylitis Disease Activity Index (BASDAI)), physical function (the Bath Ankylosing Spondylitis Functional Index (BASFI)), spinal mobility (the Bath Ankylosing Spondylitis Metrology Index (BASMI)), and r-axSpA-related radiographic spinal changes (the modified Stoke Ankylosing Spondylitis Spinal Score (mSASSS)) were evaluated using established instruments [6].

Patients of similar age as controls (50–64 years) without a history of cardiac stent or bypass surgery were invited to be examined by coronary artery calcium computed tomography (CAC CT). CAC CT was performed using a dual-source CT scanner (Siemens Somatom Force, Siemens Healthineers, Forchheim, Germany) with a tube voltage of 120 kV. A high-pitch spiral scan was used on patients with a low (≤65 bpm) and regular heart rate. A sequential acquisition was applied if the heart rate was >66 bpm or irregular. Images were reconstructed with a slice thickness of 3 mm, and CAC was scored according to the Agatston method [7]. The CAC score was calculated separately for the three coronary territories (circumflex (CX) coronary artery, the left main (LM) and left anterior descending (LAD) coronary artery combined (LM-LAD), and the right coronary artery (RCA)) and summed for a total CAC score.

Data from controls without rheumatic disease were obtained from the Swedish CArdioPulmonary bioImage Study (SCAPIS). The SCAPIS study protocol has been described in detail previously [8]. In brief, participants aged 50–64 years were recruited by random selection from the population register in six university cities in Sweden. The only exclusion criterion was the inability to provide informed consent. In total, 30,154 participants were enrolled, and in southwestern Sweden, 6589 participants were enrolled. For each patient, four controls, 232 in total, without a history of cardiac stent or bypass surgery, examined by CAC CT, were matched on age (±2 years), sex, and geographic region (Gothenburg study center). For both the patients and the controls in southwestern Sweden, CAC CT was performed at the Department of Radiology at the Sahlgrenska University Hospital.

### Statistical Analysis

To investigate differences in the presence of CAC between patients and controls, a logistic regression was used, where CAC was modeled as dichotomous (rounded to the nearest integer, i.e., CAC score > 0 versus CAC score = 0). When examining factors associated with total CAC score in patients with r-axSpA, the Jonckheere–Terpstra test of trends in medians between different cutoffs based on CAC score was applied. Total CAC score was split into a trichotomous variable (0/>0–99/≥100), and trends in medians of ASDAS, BASFI, BASMI, BASDAI, time-averaged CRP, mSASSS, symptom duration, cigarette exposure (pack years), and age were assessed. To test distributions between categorical variables, Fisher’s Exact test and Fisher-Freeman-Halton tests were used. For comparison of continuous and ranked variables between two groups, either the Wilcoxon Rank-Sum test or the *t*-test was used, depending on data distribution and variable type. Statistical analysis was completed in SAS for Windows version 9.4 and IBM SPSS version 29. Graphical presentations were created using RStudio 2024.09.1+394 for Windows. Statistical significance was determined at *p* < 0.05.

## 3. Results

**Figure 1:**
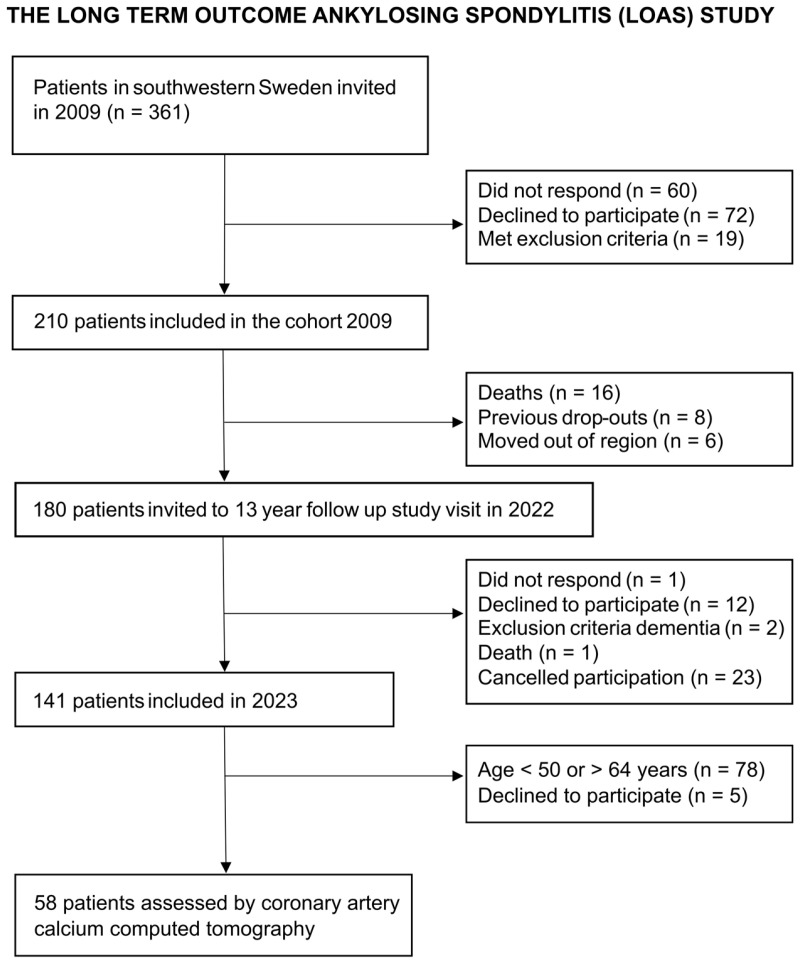
Recruitment of patients with r-axSpA assessed by coronary artery calcium computed tomography to be compared to controls.

In total, 58 patients and 232 matched controls were included (Figure 1). The median age was 58 years, and 48% were female (Table 1). A significantly higher proportion of patients had a diagnosis of hypertension, and hsCRP concentration was higher among patients compared to controls. In contrast, there were no significant differences in the proportion of previous smokers, diabetes diagnosis, or the apolipoprotein (Apo) B/A1 ratio between patients and controls.

Among the patients, 83% were human leukocyte antigen (HLA)-B27 positive, 17% were treated with conventional synthetic disease-modifying antirheumatic drugs (csDMARDs), 38% were treated with biological disease-modifying antirheumatic drugs (bDMARDs), and 29% used non-steroidal anti-inflammatory drugs (NSAIDs) at least twice per week.

### 3.1. Comparison of CAC Score in Patients with r-axSpA Versus Controls

**Figure 2:**
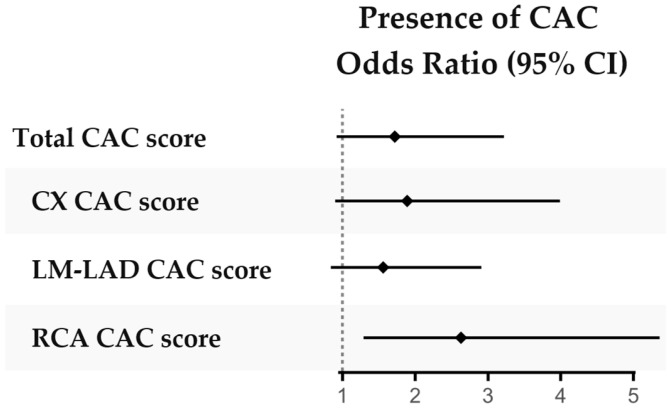
Presence of CAC in patients with r-axSpA versus controls. Presence of CAC (score = 0 versus > 0) compared between patients and controls in logistic regressions adjusted for sex, age and smoking history (ever smoked yes/no). Abbreviations: CAC coronary artery calcification, CX circumflex coronary artery, LM-LAD left main and left anterior descending coronary artery, RCA right coronary artery.

The total median CAC score did not differ significantly between patients compared to controls, although the CAC score in RCA was higher in patients (Table 1). In a logistic regression assessing the presence of detectable CAC (i.e., CAC score > 0 versus CAC score = 0), a significantly higher proportion of patients had detectable CAC in RCA compared to controls, but this was not seen in other coronary arteries (Figure 2, Supplemental Table S1).

### 3.2. Factors Associated with Higher CAC Score in Patients with r-axSpA

**Figure 3:**
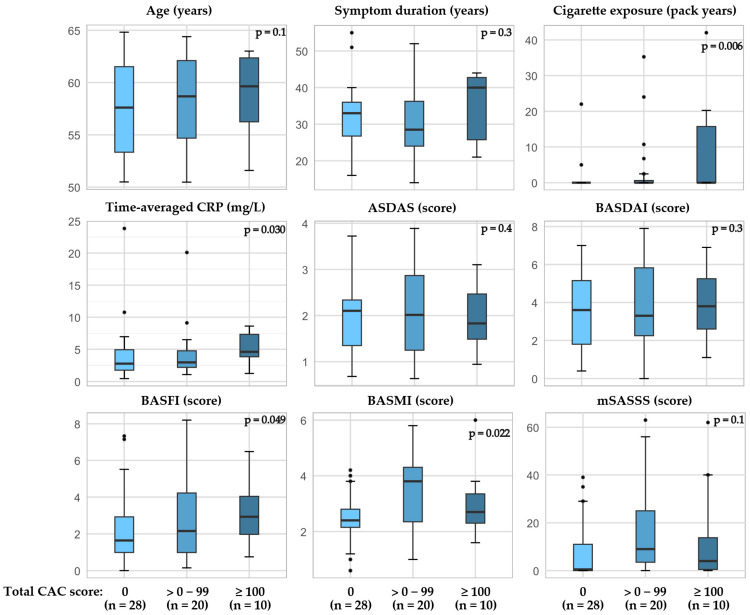
Total CAC score in patients with r-axSpA and associations with age, cigarette exposure, and disease outcomes. P-value calculated by Jonckheere-Terpstra test for trend in medians, one-sided. Abbreviations: ASDAS the Ankylosing Spondylitis Disease Activity Score with CRP, BASFI the Bath Ankylosing Spondylitis Functional Index, BASMI the Bath Ankylosing Spondylitis Metrology Index, CAC coronary artery calcification, CRP C-reactive protein, mSASSS modified stoke ankylosing spondylitis spinal score.

**Figure 4:**
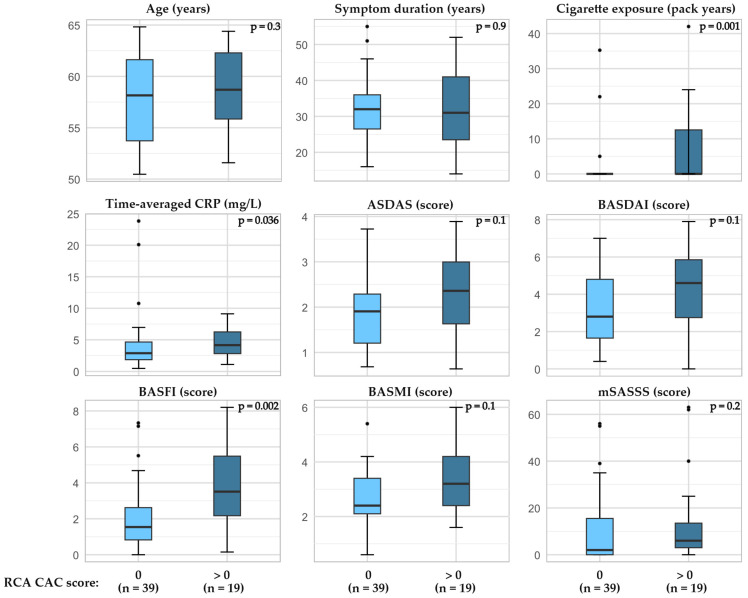
Presence of CAC in RCA and relation to age, cigarette exposure, and disease outcomes in patients with r-axSpA. P-value calculated by Wilcoxon Rank-Sum test, exact two-tailed significance value. Abbreviations: ASDAS the Ankylosing Spondylitis Disease Activity Score with CRP, BASFI the Bath Ankylosing Spondylitis Functional Index, BASMI the Bath Ankylosing Spondylitis Metrology Index, CAC coronary artery calcification, CRP C-reactive protein, mSASSS modified stoke ankylosing spondylitis spinal score, RCA right coronary artery.

In patients with r-axSpA, higher a BASFI and BASMI as well as time-averaged CRP and cigarette exposure (pack years) were associated with higher total CAC scores (Figure 3, Supplemental Table S2). Furthermore, when examining factors related to the presence of CAC in the RCA, the BASFI score as well as time-averaged CRP and cigarette exposure were higher in patients with CAC compared to the patients without CAC (Figure 4, Supplemental Table S3). The CAC score did not appear to be related to bDMARD or regular NSAID usage (Supplemental Table S2).

## 4. Discussion

This study was set up to investigate the prevalence and severity of CAC in patients with r-axSpA compared to controls without r-axSpA, as well as the association between disease outcomes and CAC score in patients. We found that the total CAC score was not significantly different between patients and controls but that the prevalence of CAC in RCA was higher in patients and that a decrease in spinal mobility and physical function, time-averaged CRP, and smoking were associated with CAC in patients with r-axSpA.

Previous data on CAC in patients with r-axSpA are scarce. Rueda-Gotor et al. [9] compared the validity of the systematic coronary risk evaluation (SCORE) [10] in comparison to CAC score and carotid intima media thickness and plaques by ultrasound examination in patients with axSpA. This investigation found that the SCORE method had inferior sensitivity to detect high-cardiovascular-risk patients based on CAC score and ultrasound examination. Furthermore, non-calcified plaque in the carotid was found in 32% of patients with a CAC score of 0, indicating that these methods detect different processes of atherosclerotic development. Overall, relying on traditional risk stratification may thus underestimate the CVD risk in patients with r-axSpA. Studies on the utility of risk prediction algorithms for CVD in r-axSpA appear to be scarce [11].

An increased coronary atherosclerotic plaque burden has been described in younger patients with r-axSpA compared to controls [12], and an increased carotid intima media thickness as well as arterial stiffness [13] has been found in patients with r-axSpA compared to controls. While these findings are indicative of increased CVD risk in this patient group, our observation of increased CAC score in RCA represents, to the best of our knowledge, a novel finding. While the total CAC score is generally used in risk stratification for CVD, a decreased circulation in RCA might be of particular interest due to the potential impact on the sinoatrial and atrioventricular nodes, which are critical parts of the electrical conduction system of the heart. Although speculative, this finding might be an explanatory factor for the increased prevalence of cardiac rhythm disturbances seen in patients with r-axSpA, as compared to the general population [14]. However, this hypothesis needs to be studied in a larger patient sample before any conclusions can be drawn.

The association of time-averaged CRP with CAC scores indicates the importance of monitoring inflammation and securing optimal disease management in patients with r-axSpA. While effective treatment has been increasingly available in the past decades, presumably leading to improved inflammatory status, elevated CRP levels remain a significant risk factor for CVD development in patients with r-axSpA [15]. In our cohort, CRP at the latest follow-up was still significantly higher than that seen in controls, and a higher proportion of patients had elevated CRP concentrations (>3 mg/L), suggesting a persisting chronic low-grade inflammation in some of the patients even in the era of modern anti-rheumatic drugs.

Spinal mobility impairment (BASMI), often a result of chronic inflammation and an effect of prolonged disease activity in patients with r-axSpA, was also associated with a higher total CAC score. While this association warrants further investigation, one plausible explanation for this finding could be its relation to historical inflammation. Similarly, impairment in physical function (BASFI) was associated with both the total CAC score and CAC in RCA. A low level of physical function, like the BASMI, might be related to prolonged disease activity as well as lower ability to partake in physical exercise, which again could accelerate CVD development.

As recently reviewed, traditional risk factors such as hypertension, obesity, metabolic syndrome, and cigarette smoking may be more prevalent in patients with r-axSpA compared to controls [16]. In our cohort, hypertension was more prevalent in patients compared to controls but not BMI, diabetes, or history of smoking. The impact of smoking on CAC score has been clearly demonstrated previously [17]. Our findings of a significant association between cigarette exposure and CAC score in patients with r-axSpA further highlight the important effect of lifestyle factors on health outcomes in this patient group.

### Strengths and Limitations

Our study has limitations that merit mentioning. Our results are limited to patients aged 50–64 years old and with a long-standing disease duration. The limited sample size elevates the risk of type-2 errors and restricts the number of covariates to adjust for in regression models without violating model assumptions. We, furthermore, did not have a detailed variable for physical activity in patients and controls; thus, we could not adjust for this factor. Most of the comparisons are also performed as cross-sectional analyses, which renders causal interpretations inappropriate.

There are also several strengths in this investigation. The original patient cohort initially invited all patients with a verified r-axSpA diagnosis that fulfilled the inclusion criteria and did not meet the exclusion criteria. Patients of appropriate age to be matched to controls were then invited to this sub-study. The controls were taken from the SCAPIS study, which invited participants in the age range of 50–64 years old by random selection from the population register, thus increasing the likelihood of a control sample representative of the general population. We applied appropriate regression models adjusted for key confounders, and the CAC scores of both patients and controls were assessed at the same outpatient clinic. The collection of CRP concentrations over time, instead of using a single potentially transient value, opens the possibility of drawing causal conclusions about the effects of chronic inflammation in this patient group.

## 5. Conclusions

Chronic inflammation appears to accelerate coronary calcification and thereby predispose CVD development in patients with r-axSpA, indicating the importance of efficient treatment to reduce systemic inflammation. Furthermore, decreased spinal mobility and impaired physical function were related to coronary calcification, which warrants further investigation. While the presence of calcification in RCA was higher in patients compared to controls, no significant difference in the total CAC score was detected. However, the number of patients was relatively low, which calls for a cautious interpretation.

## Figures and Tables

**Table 1:** Descriptive data of patients with r-axSpA and matched controls.

	Patients, n = 58 Median (25p, 75p) or N (%)	Controls, n = 232 Median (25p, 75p) or N (%)	*p*-Value ^1^
Age (year)	58.4 (54.4, 62.0)	57.9 (53.9, 60.8)	0.3
Sex (male)	30 (52)	120 (52)	1.0
Body mass index (kg/m^2^)	26.8 (23.9, 29.3)	26.4 (23.8, 28.9)	0.6
HLA-B27+	48 (83)	-	
ASDAS (score)	2.02 (1.32, 2.57)	-	
BASDAI (score)	3.5 (1.8, 5.63)	-	
BASFI (score)	2.6 (2.2, 3.8)	-	
BASMI (score)	3.5 (1.8, 5.63)	-	
mSASSS (score)	4 (0, 16.3)	-	
ESR (mm/h)	11 (5.8, 18)	-	
hsCRP (mg/L)	1.7 (1.1, 3.7)	0.98 (0.5, 2.1)	0.001
hsCRP > 3 mg/L	17 (29)	41 (18)	0.040
Time-averaged CRP (mg/L)	3.0 (1.9, 5.2)	-	
Symptom duration (years)	32 (24, 38)	-	
Hypertension diagnosis ^2^	22 (39)	50 (22)	0.016
Diabetes diagnosis ^2^	2 (4)	6 (3)	0.6
Ever been a smoker	26 (45)	130 (56)	0.1
Apo B/A1 (ratio)	0.6 (0.5, 0.7)	0.6 (0.5, 0.7)	0.2
NSAID usage >2 times/w ^3^	17 (29)	-	
csDMARD	10 (17)	-	
bDMARD	22 (38)	-	
Total CAC score grading			0.4
None (<1)	29 (50)	137 (59)	
Minimal/mild (1–99)	19 (33)	66 (28)	
Moderate (≥100)	10 (17)	29 (13)	
Total CAC score	0.5 (0, 73.5)	0 (0, 19)	0.2
Mean ± SD	82.6 ± 220	49.8 ± 162	
CX	0 (0, 0.3)	0 (0, 0)	0.1
Mean ± SD	16.3 ± 64.7	7.6 ± 42.2	
LM-LAD	0 (0, 20.3)	0 (0, 0)	0.3
Mean ± SD	52.5 ± 145	29.3 ± 84.1	
RCA	0 (0, 3.3)	0 (0, 0)	0.036
Mean ± SD	13.8 ± 40.6	12.9 ± 74.2	

^1^ *p*-values for continuous variables calculated by Wilcoxon Rank-Sum test using exact two tailed significance; for categorical variables, the Fisher’s Exact test or the Fisher–Freeman–Halton Exact Test was used. ^2^ Data missing for one patient and one control. ^3^ Data missing for two patients. Abbreviations: Apo—apolipoprotein, ASDAS—the Ankylosing Spondylitis Disease Activity Score with CRP, BASFI—the Bath Ankylosing Spondylitis Functional Index, BASMI—the Bath Ankylosing Spondylitis Metrology Index, bDMARD—biological disease-modifying antirheumatic drug, CRP—C-reactive protein, csDMARD—conventional synthetic disease-modifying antirheumatic drug, HLA—human leukocyte antigen, mSASSS—modified stoke ankylosing spondylitis spinal score, CAC score—coronary artery calcification score, CX—circumflex coronary artery, hsCRP—high-sensitivity C-reactive protein, ESR—erythrocyte sedimentation rate, LM-LAD—left main and left anterior descending coronary artery, NSAID—non-steroidal anti-inflammatory drug, RCA—right coronary artery.

## Data Availability

Due to Swedish privacy laws, data are not shared publicly; however, a limited and fully anonymized dataset that supports the main analyses is available upon reasonable request from the corresponding authors.

## Supplementary Materials

The following supporting information can be downloaded at: https://www.mdpi.com/article/10.3390/jcdd12080305/s1, Supplemental Table S1: Comparison of presence of CAC between patients with r-axSpA and matched controls in a logistic regression; Supplemental Table S2: Total CAC score in patients with r-axSpA and associations with age, smoking and disease outcomes; Supplemental Table S3: Presence of CAC in RCA and relation to age, cigarette exposure and disease outcomes in patients with r-axSpA.

## Abbreviations

The following abbreviations are used in this manuscript:

Apo	Apolipoprotein
ASDAS	Ankylosing Spondylitis Disease Activity Score with CRP
BASDAI	Bath Ankylosing Spondylitis Disease Activity Index
BASFI	Bath Ankylosing Spondylitis Functional Index
BASMI	The Bath Ankylosing Spondylitis Metrology Index
bDMARD	Biological disease-modifying antirheumatic drug
CAC	Coronary artery calcification
CRP	C-reactive protein
csDMARD	Conventional synthetic disease-modifying antirheumatic drug
CT	Computed tomography
CVD	Cardiovascular disease
CX	Circumflex
ESR	Erythrocyte sedimentation rate
HLA	Human leukocyte antigen
hsCRP	High-sensitivity C-reactive protein
LAD	Left anterior descending coronary artery
LM	Left main coronary artery
LM-LAD	The left main and left anterior descending coronary artery
LOAS	Long-term Outcome Ankylosing Spondylitis
mSASSS	Modified Stoke Ankylosing Spondylitis Spinal Score
NSAID	Non-steroidal anti-inflammatory drug
r-axSpA	Radiographic axial spondyloarthritis
RCA	Right coronary artery
SCAPIS	Swedish CArdioPulmonary BioImage Study
