# Supplementary material for: Coronary Artery Calcification in Patients with Radiographic Axial Spondyloarthritis: A Comparative Study with Matched Controls in Southwestern Sweden
Source: J Cardiovasc Dev Dis. 2025 Aug 12;12(8):305. doi: 10.3390/jcdd12080305 (PMC12387049; doi:10.3390/jcdd12080305)
Supplement: Supplementary file 1 [file jcdd-12-00305-s001.zip › jcdd-3703615-supplementary.pdf]

Supplemental Table S1. Comparison of presence of CAC between patients with r-axSpA and matched controls in a logistic regression

	Total CAC score		CX		LM-LAD		RCA	
	**OR (95% CI)**	**p-value**	**OR (95% CI)**	**p-value**	**OR (95% CI)**	**p-value**	**OR (95% CI)**	**p-value**
Case (yes/no)	1.72 (0.92-3.22)	0.089	1.89 (0.90-3.99)	0.094	1.56 (0.84-2.91)	0.164	2.63 (1.29-5.36)	0.008
Age (years)	1.07 (1.003-1.14)	0.039	1.10 (1.01-1.20)	0.030	1.05 (0.98-1.12)	0.144	1.10 (1.01-1.19)	0.025
Sex (male)	3.70 (2.22-6.17)	< 0.001	4.15 (1.99-8.64)	< 0.001	3.76 (2.25-6.29)	< 0.001	3.95 (2.04-7.65)	< 0.001
Never smoked	0.49 (0.29-0.81)	0.006	0.75 (0.39-1.45)	0.391	0.54 (0.32-0.89)	0.017	0.34 (0.18-0.65)	0.001

Abbreviations: CAC Coronary artery calcification, CX circumflex coronary artery, LM-LAD the left main and left anterior descending coronary artery, OR Odds ratio, RCA right coronary artery.

Supplemental Table S2. Total CAC score in patients with r-axSpA and associations with age, cigarette exposure and disease outcomes

	CAC score = 0 (n = 28) Median (25p, 75p) or N(%)	CAC score > 0 – 99 (n = 20) Median (25p, 75p) or N (%)	CAC score ≥100 (n = 10) Median (25p, 75p) or N (%)	P-value[1]
**Age (years)**	58 (53, 62)	59 (55, 62)	60 (56, 62)	0.1
**ASDAS (score)**	2.1 (1.3, 2.4)	2.0 (1.2, 2.9)	1.8 (1.5, 2.5)	0.4
**BASDAI (score)**	3.6 (1.8, 5.3)	3.3 (1.9, 5.9)	3.8 (2.5, 5.3)	0.3
**BASFI (score)**	1.6 (0.9, 2.9)	2.2 (1.0, 4.4)	2.9 (1.9, 4.2)	0.049
**BASMI (score)**	2.4 (2.1, 2.8)	3.8 (2.3, 4.4)	2.7 (2.2, 3.4)	0.022
**mSASSS (score)**	0.5 (0.0, 12.0)	9.0 (3.0, 25.0)	4.0 (0.0, 17.0)	0.1
**Cigarette exposure (pack years)**	0.0 (0.0, 0.0)	5.6 (0.0, 1.2)	4.5 (0.0, 16.2)	0.006
**Symptom duration (years)**	33 (27, 36)	29 (24, 37)	40 (24, 43)	0.3
**Time-averaged CRP (mg/L)**	2.8 (1.7, 5.4)	3.0 (2.2, 4.9)	4.6 (3.4, 7.8)	0.030
**bDMARD**	11 (39)	7 (35)	4 (40)	1.0
**NSAID usage >2 times/w[2]**	6 (22)	9 (45)	2 (22)	0.2

[1] Jonckheere-Terpstra test for trend in medians, one-sided. Fisher Freeman Halton Exact test for comparisons of proportions of participants per group.

[2] Data is missing from 2 patients.

Abbreviations: ASDAS the Ankylosing Spondylitis Disease Activity Score with CRP, BASFI the Bath Ankylosing Spondylitis Functional Index, BASMI the Bath Ankylosing Spondylitis Metrology Index, bDMARD biological disease modifying antirheumatic drug, CAC score coronary artery calcification score, CRP C-reactive protein, mSASSS modified stoke ankylosing spondylitis spinal score, NSAID Non-steroidal anti-inflammatory drug.

Supplemental Table S3. Presence of CAC in RCA and relation to age, cigarette exposure and disease outcomes in patients with r-axSpA

	**RCA CAC score = 0 (n = 39) Median (25p, 75p)**	**RCA CAC score > 0 (n = 19) Median (25p, 75p)**	**P-value[1]**
**Age (years)**	58.2 (53.6, 61.8)	58.7 (55.6, 62.4)	0.3
**ASDAS (score)**	1.9 (1.2, 2.3)	2.4 (1.5, 3.1)	0.1
**BASDAI (score)**	2.8 (1.5, 5.0)	4.6 (2.6, 5.9)	0.1
**BASFI (score)**	1.5 (0.7, 2.9)	3.5 (2.1, 6.1)	0.002
**BASMI (score)**	2.4 (2.0, 3.8)	3.2 (2.2, 4.6)	0.1
**mSASSS (score)**	2.0 (0.0, 16.0)	6.0 (2.0, 17.0)	0.2
**Cigarette exposure (pack years)**	0.0 (0.0, 0.0)	0.0 (0.0, 14.4)	0.001
**Symptom duration (years)**	32.0 (26.0, 36.0)	31.0 (23.0, 42.0)	0.9
**Time-averaged CRP (mg/L)**	2.9 (1.8, 4.7)	4.2 (2.7, 6.5)	0.036

[1] Wilcoxon Rank-Sum test, two-tailed exact significance.

Abbreviations: ASDAS the Ankylosing Spondylitis Disease Activity Score with CRP, BASFI the Bath Ankylosing Spondylitis Functional Index, BASMI the Bath Ankylosing Spondylitis Metrology Index, CAC score coronary artery calcification score, CRP C-reactive protein, mSASSS modified stoke ankylosing spondylitis spinal score, RCA right coronary artery.
